# Impacts of the Finnish service screening programme on breast cancer rates

**DOI:** 10.1186/1471-2458-8-38

**Published:** 2008-01-28

**Authors:** Ahti Anttila, Tytti Sarkeala, Timo Hakulinen, Sirpa Heinävaara

**Affiliations:** 1 Mass Screening Registry, Finnish Cancer Registry, Helsinki, Finland; 2 Finnish Cancer Registry, Helsinki, Finland; 3 Research and Environmental Surveillance, STUK Radiation and Nuclear Safety Authority, Helsinki, Finland

## Abstract

**Background:**

The aim of the current study was to examine impacts of the Finnish breast cancer (BC) screening programme on the population-based incidence and mortality rates. The programme has been historically targeted to a rather narrow age band, mainly women of ages 50–59 years.

**Methods:**

The study was based on the information on breast cancer during 1971–2003 from the files of the Finnish Cancer Registry. Incidence, cause-specific mortality as well as incidence-based (refined) mortality from BC were analysed with Poisson regression. Age-specific incidence and routine cause-specific mortality were estimated for the most recent five-year period available; incidence-based mortality, respectively, for the whole steady state of the programme, 1992–2003.

**Results:**

There was excess BC incidence with actual screening ages; incidence in ages 50–69 was increased 8% (95 CI 2.9–13.4). There was an increasing temporal tendency in the incidence of localised BC; and, respectively, a decrease in that of non-localised BC. The latter was most consistent in age groups where screening had been on-going several years or eventually after the last screen. The refined mortality rate from BC diagnosed in ages 50–69 was decreased with -11.1% (95% CI -19.4, -2.1).

**Conclusions:**

The current study demonstrates that BC screening in Finland is effective in reducing mortality rates from breast cancers, even though the impact on the population level is smaller than expected based on the results from randomised trials among women screened in age 50 to 69. This may be explained by the rather young age group targeted in our country. Consideration whether to targeted screening up to age 69 is warranted.

## Background

Impact of mammography screening in decreasing breast cancer (BC) mortality has been shown among women invited in ages 50–69 in several randomised trials [1]. There is growing evidence from cohort follow-up studies that the service screening programmes implemented in the late 1980s or early 1990s affect breast cancer mortality among invited with at least a similar degree than the randomised trials [2-6]. Studies using dynamic materials [7-9] or modelling historical screening coverage [10,11] have also demonstrated effectiveness of organised screening. On the other hand, there are proposals that screening has not clearly affected population breast cancer mortality rates [12,13] or, breast cancer or overall mortality is not affected in general [14,15].

There are several biases in the dynamic population-based studies. Death from breast cancer occurs often several years after the diagnosis; studies based on routine deaths records may suffer from misclassification of the screening status [2,7,12]. This can be corrected if incidence-based mortality is used. Another source of bias in the early trend studies is that national screening programmes have been introduced gradually, and the screening coverage in the targeted groups had become complete only after several years of implementation.

The aim of the current study was to examine impacts of the breast cancer screening programme in Finland on the population-based incidence and mortality rates. Finland makes an exceptional setting for the study, because the programme has been targeted historically to a rather narrow age band mainly among women of ages 50–59 years.

## Methods

The study was based on the information on breast cancer (ICD-10 code C50) from the files of the Finnish Cancer Registry. The women diagnosed with a new (incident) primary breast cancer (N = 74,175), or died from breast cancer (N = 22,799), in 1971–2003 were included. Analysis was restricted to invasive breast cancers. Following targeted age groups in screening, ages 40–69 were included in the analysis of incidence data; follow-up of subsequent deaths from breast cancer was extended up to age 79. The incident cases were classified into two groups by stage of the disease, localised and non-localised at diagnosis, based on information mainly on the lymph node status as available in the cancer registration. Among 9.4% of the BC cases the stage information was not available at the cancer registry. The proportion cases with stage unknown was 13% in 2003 and thus stage-specific information was considered adequate for the analyses up to the year 2002.

In order to assess historical coverage of screening, national data on the invitations were collected from the records of the Mass Screening Registry – a subunit of the Finnish Cancer Registry. Coverage of the screening registration is variable during the 15 years' period of routine BC screening: in 1987–1990 the average coverage of registration of invitations is 83% (N = 358,200/431,300) whereas from 1991 onwards the estimated annual registration coverage is > 95% (1,257,000/1,259,200; the estimated average registration coverage 99.8%). These estimates are derived from comparisons of the yearly registered invitations with an external documentation on the invitations, maintained by the Radiological Society of Finland [16].

If a municipality or a screening centre had not registered their invitations, we requested from the municipality health authorities or screening centres mailed information on invited age groups, by birth cohort and invitational year, and estimated thereafter the biannual coverage of invitations by combining numbers of invited with the respective population data. We obtained mailed information on screening invitations during 1987–1990 for 140 municipalities for which there were registered invitational data available only from 1991 onwards. During the overall study period the number of municipalities changed from 461 (1987, 1988) to 446 (2002); annual invitational data became available in total for 410 (89%) of them.

For incidence-based, i.e., refined mortality, individual patient data were studied. Only those deaths were included, where the diagnosis of breast cancer took place in the given calendar period and age group. For the refined mortality, the age was defined as the age at diagnosis of the corresponding incident case. Deaths from breast cancer were included and stratified by the 5-year calendar period, birth-cohort, and age at diagnosis. In these data, breast cancer deaths taking place at an older age than 79 years were excluded. The length of follow-up after diagnosis was made symmetric between the groups on age and period: Deaths in 1972–1983 among those diagnosed in 1972–76 in ages 40 and 69, deaths in 1977–88 among those diagnosed in 1977–81 in ages 40–69, and so on, until deaths in 1992–2003 among those diagnosed in 1992–1996. There was thus a 5-year long period for incidence and a 12-year long period for mortality in each time window. Incident cases from the calendar period 1992–1996 were the most recent ones included in this incidence-based analysis. This restriction was done to obtain comparability of information over the decades, and to obtain also as long follow-up time as possible.

Annual population denominators were used by 1-year age group for estimating screening coverage, as well as in estimating refined mortality follow-up; and by 5-year age group by calendar year for breast cancer incidence and routine mortality rates.

### Coverage estimates of screening

**Figure 1:**
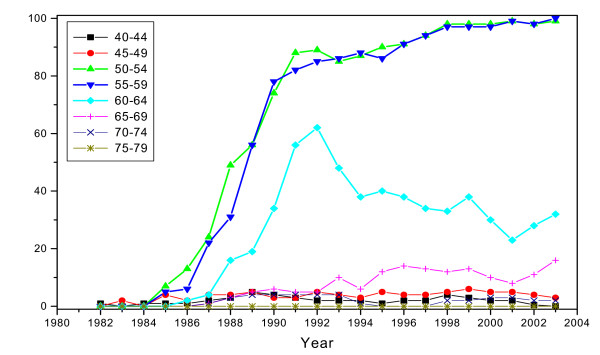
Estimated biannual coverage of invitations in the national breast cancer screening programme in Finland in 1982–2003, by age.

Invitational coverage was defined as the number of women invited at least once within the recommended screening interval (a two-year period) per population size. The actual invitational coverage increased gradually during the first five years of the programme, 1987–1991; i.e., during the pseudo-randomised implementation phase (Figure 1). The actual coverage proportions were converted to estimates describing invitation (yes/no) ever in lifetime. Estimated coverage of 'ever' invited was approximated within ages 50–64 at 10%–89% during 1987–1991, and 100% since 1992. In ages 65–69, lifetime coverage increased over 10% since 1992 and up to 100% since 1997; mainly while ageing of the invited group.

### Statistical analyses

The incidence of and mortality from breast cancer as well as the incidence-based mortality were analysed with Poisson regression. To adjust for the fluctuating changes in the incidence and mortality with age and cohort, the incidence and mortality were modelled as functions of numerical calendar year and polynomials of numerical age at 5-year age groups and synthetic birth cohort [17]. The orders of polynomials i.e., the forms of marginal age and cohort curves were searched for overall incidence, localised and non-localised incidence, and mortality separately using the data of calendar period 1953–1970. The decision of the chosen degrees of polynomials was based on the likelihood ratio statistic and descriptive evaluation. In the estimation of overall incidence, 10th order polynomial of age and 4th order polynomial of cohort were needed, in localised and non-localised incidence the corresponding orders of age were 10 and 6, and of cohort 8 and 2, respectively, and in mortality the orders of polynomials were 5 for both age and cohort. In the analysis of incidence-based mortality, the orders of polynomials for age at diagnosis and cohort were both 3, and each year in the follow-up between the diagnosis and death was included as a level of categorical variable.

In the statistical analyses we paid emphasis on the steady state of the programme with a high actual screening coverage among women in the main target ages (particularly, ages 50–59). We included an indicator for the period from 1992 onwards – with the exclusion of years 1987–91 – indicating the steady state of the screening programme. During the steady state, prevalence screens took place mainly at the onset at age 50. In the first descriptive phase, observation during the recent screening period 1998–2002 was contrasted with expectation without screening as drawn from modelling information prior to the screening era (years 1971–1986) within each age group. Because of the symmetry requirements in the data input when studying incidence-based mortality (see above), this expectation could not be estimated for the completely same period of time and these results are shown separately. In the second phase, the effect of screening was studied for the screened age groups only (with the estimated coverage at least 90%). For example, the age group 60–64 years consisted of screened birth-year cohorts after 1992, and the groups 65–69 years since 1997. In order to estimate screening effects, parallel developments among screened and non-screened were assumed. Non-screened group consisted here of women in ages 40–49 -years; and ages 65–69 years up to the end of 1991. Note that even the observed incidence and mortality rates shown below in the Tables are based on models.

### Ethical consideration

Information on the breast cancer incidence and mortality, as well as on the population numbers and screening indices were based on tabular statistical data only; according to the current legislature, no approval from the ethical committee was required.

## Results

**Figure 2:**
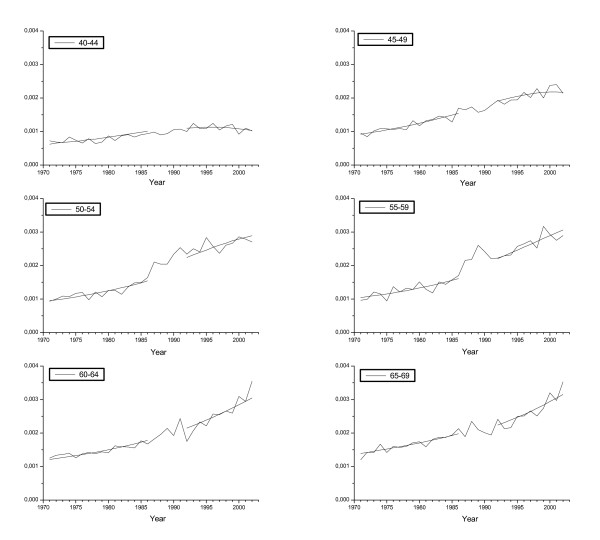
Observed and fitted breast cancer incidence rates by age group. The first five years since the implementation of the national programme have been excluded from the fitted rates.

**Figure 3:**
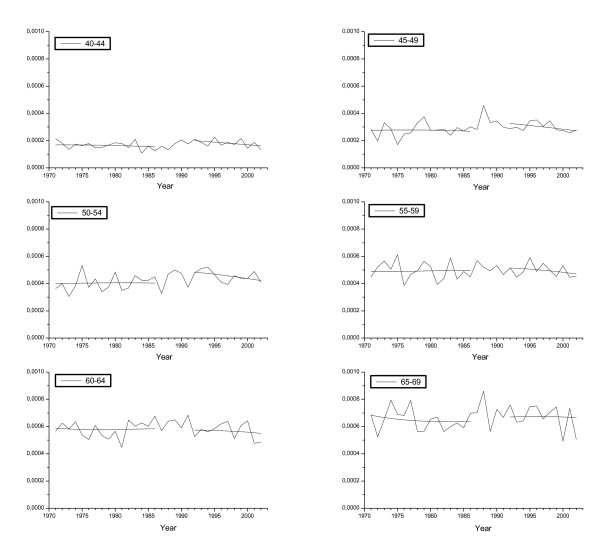
Observed and fitted breast cancer mortality rates by age group at death. The first five years since the implementation of the national programme have been excluded from the fitted rates.

Table 1 shows age-specific BC incidence and mortality rates observed in 1998–2002; corresponding estimates without screening as extrapolated from mortality experience in these same age groups from time before national implementation of screening; excess relative risk estimates; and woman-years at risk. In addition, Figures 2 and 3 illustrate the overall developments of BC incidence and routine mortality trends by calendar year and 5-year age group among women in ages 40–69; as well as the values obtained by fitting the models.

**Table 1:** Woman-years at risk, and observed and expected incidence and mortality rates (/100) of breast cancer in Finland in 1998–2002 by age group. The expected rates are based on an extrapolation of models fitted in the data during 1971–1986 (before screening era).

	Age group (years)							
	40–44	45–49	50–54	55–59	60–64	65–69	40–69	50–69
Woman-years at risk	943,300	992,500	1,034,400	788,500	680,900	614,900	5,054,500	3,118,700
								
*Incidence, any*								
Observed with screening	0.108	0.217	0.279	0.290	0.285	0.294	0.239	0.286
Expected without screening^1^	0.113	0.211	0.236	0.261	0.288	0.309	0.237	0.262
Excess relative risk (in %)^1^	-4	3	18*	11*	-1	-5	1	9*
								
*Incidence, localised at diagnosis*								
Observed with screening	0.054	0.119	0.168	0.182	0.182	0.181	0.143	0.177
Expected without screening^1^	0.045	0.095	0.128	0.131	0.158	0.173	0.116	0.141
Excess relative risk (in %)^1^	20*	26*	56*	39*	15*	5	23*	26*
								
*Incidence, non-localised at diagnosis*								
Observed with screening	0.056	0.093	0.098	0.092	0.092	0.104	0.088	0.097
Expected without screening^1^	0.071	0.100	0.105	0.112	0.117	0.124	0.105	0.106
Excess relative risk (in %)^1^	-21*	-7	-6	-18*	-21*	-16*	-16*	-9*
								
*Mortality*								
Observed with screening	0.017	0.028	0.044	0.048	0.056	0.067	0.041	0.052
Expected^1 ^without screening	0.012	0.022	0.035	0.046	0.057	0.064	0.037	0.051
Excess relative risk^1 ^(in %)	42*	29*	24	5	-2	4	10	3

^1 ^(Observed – expected)/expected * Significant at 0.05 level

There was excess BC incidence with actual screening ages (18% and 11% in ages in age 50–54 and 55–59 years, Table 1). Among all age groups studied, there was an increasing tendency of localised breast cancers, particularly pronounced in screening target ages; and, respectively, a decreasing trend in incidence of non-localised breast cancers. The decrease in the incidence of non-localised breast cancers was most consistent in ages 55 to 69 years, i.e. in late screening or up to five years after the last screen.

Age-specific routine breast cancer mortality rate had increased up to mid-1990s particularly in ages younger than targeted by the national screening programme (ages 40–44, and 45–49 years; Table 1). We observed a consistent decreasing trend in the mortality rate since the mid-1990s among all studied age groups, however (Figure 3). In general, the death rates in ever-screened age groups (50 to 69, age at death) had increased less than among non-screened or remained stable (Table 1).

Incidence-based mortality rates from breast cancers diagnosed in ages 40–49 showed an increase of 28%–34%. In screening ages (50 to 69), the corresponding point estimates showed slightly smaller increase or a decreasing tendency (Table 2). The decrease was largest in deaths from BC cases which had been diagnosed in ages 55–59 and 60–64. Specific to the age groups, the incidence-based mortality result was statistically significant only among women of ages 45–49.

**Table 2:** Observed and expected incidence-based mortality (/100) in 1992–2002 from breast cancers diagnosed in 1992–96 and the modelled changes in the incidence-based mortality from breast cancers diagnosed in the three five-year periods during 1972–1986 to 1992–1996, by age at diagnosis.

	Age group at diagnosis (years)							
	40–44	45–49	50–54	55–59	60–64	65–69	40–69	50–69
Observed	0.005	0.008	0.011	0.009	0.011	0.013	0.009	0.011
Expected ^1^	0.004	0.006	0.010	0.010	0.012	0.012	0.009	0.011
Excess relative risk (in %)^1^	34	28 *	11	-7	-11	11	4	-3

^1 ^(Observed – expected)/expected * Significant at 0.05 level

Table 3 summarises the tentative screening effects, obtained from comparisons of trends between screened and non-screening age groups. The overall BC incidence was in excess of 8.0%; particularly the incidence of localised BCs was in excess (22.5%). Incidence of non-localised breast cancers at diagnosis was 9.0% lower than the expectation if absence of screening. Routine BC mortality in all the screened ages combined (age at death) had decreased with -5.6% as compared with expectation without screening. Change in refined BC mortality was -11.1% (95% CI -19.4, -2.1%), respectively. Among women screened most intensively (50–64) the corresponding estimates by 5-year age groups were -3.1 %, -14.7% and -17.1%, respectively.

**Table 3:** Excess relative risks of incidence of and mortality from breast cancer in 1992–2002, and of incidence-based mortality in 1992–2002 in comparison with the baseline without screening by age. The baseline without screening was estimated by models using information from time before screening and in age groups not subjected for service screening (40–49; and 65–69 up to the year 1991, see the text).

	Excess relative risk (%) by age group (years) ^A^						
	50–54	55–59	60–64	65–69	50–59	60–69	50–69
Incidence	19.1(12.0,26.5)	11.5(4.3,19,3)	2.0(-5.0,9.4)	-2.2(-8.7,4.8)	15.9(9.9,22.3)	1.1(-4.7,7.3)	8.0(2.9,13.4)
Loc incidence	40.9(29.2,53.6)	31.8(19.3,45.6)	10.6(-0.5,22.8)	2.8(-7.5,14.3)	37.8(27.5,48.8)	8.6(-0.8,18.8)	22.5(14.2, 31.3)
Non-loc. incidence	2.5(-5.9,11.7)	-13.0(-20.5,-4.8)	-17.8(-25.2,-9.6)	-15.2(-23.6,-5.9)	-4.8(-11.2,2.1)	-15.8(-22.4,-8.7)	-9.0(-14.6,-3.2)
Mortality	1.9(-10.2,15.7)	-10.8(-22.1,2.3)	-10.9(-21.9, 1.7)	-12.1(-23.0,0.4)	-3.5(-13.6,7.9)	-9.2(-18.8,1.6)	-5.6(-14.4,4.0)
Incidence-based mortality	-3.1(-14.5,9.9)	-14.7(-24.8,-2.0)	-17.1 (-28.0,-4.7)	NA	-8.6(-17.8,1.7)	-16.6(-25.0,-4.2) ^B^	-11.1(-19.4,-2.1) ^C^

^A ^Age at diagnosis was used for the estimates for BC incidence and incidence-based mortality; age at death was used for the BC mortality.

^B ^Includes ages 60–64 at diagnosis. Excess relative risk is not available for age group 65–69 since for them the steady state started in 1997.

^C ^Includes ages 50–64 at diagnosis.

## Discussion

Two phases of analysis were constructed. The first, descriptive phase (Tables 1 and 2) gave an overall picture of the situation, whereas the second, more analytic phase (Table 3), targeted directly at estimation of the effects of the screening programme. In association with the steadily running breast cancer screening service, we observed a slight increase in the overall breast cancer incidence, and a decrease in the incidence of non-localised breast cancers. Decrease in the incidence of non-localised breast cancers may be considered an indicator predicting reductions in the mortality rate. A small even though statistically non-significant decrease was documented in the breast cancer mortality among the invited age groups. The effect of screening as obtained from incidence-based mortality analysis varied at -3.1 – -17.1% in the most intensively screened age groups. Despite the higher than predicted mortality, we observed a consistent recent decrease in breast cancer mortality since the mid-1990s in all the studied age groups 40–69.

The invitational coverage of the screening programme was close to 100% in the major targeted ages of the organised screening programme, 50–59 years of age; among non-targeted only a very low coverage was observed. We made efforts to assess coverage of the registered invitations using external information. Attendance (compliance) rates in the programme have been very high; during 1991–1999 at 89% at first screen and at 92% at subsequent screens as reported from 10 centres constituting 55–60% of the screenings of the whole national programme [18]. The Finnish Radiological Society [16] reported an average compliance rate of 89% among all the centres in 1987–1997, respectively.

In the implementation period there was a randomised screening design, affecting the population-based coverage. After 1991 (after the randomisation period) some municipalities might have got a three-year invitational interval instead of the recommended two-years for few birth cohorts. Sometimes the first screen could take place at age 51. All these affect obtaining less than full 100% age-specific coverage. After all, these deficits in screening coverage had only a minor impact in the national programme.

When considering ages targeted for screening, one limitation was that the screening coverage was not complete in ages 60–69. Another limitation was that there had been during the late 1990s another population-based invitational screening modality than the organised programme in several municipalities in this age group, paid by the women themselves. There is not much information available on the functioning of the self-paid modality. According to the only published information on a nation-wide basis, invitational coverage of this modality in ages 60–69 could have been almost at the same level as of organised screening modality during late 1990s [19]. Based data from Turku city, attendance rates in the self-paid modality could have been about 20% lower among women in age 60 to 69 than in the organised screening modality that is paid by the municipalities [20].

In the randomised screening trials an average effect of about -25% has been reported in the refined mortality rate among invited, when the screening programme has been run in ages 50–69 [1]. The current study indicated an average effect of -11.1% in the population-based refined mortality rates by respective age groups. Effects associated with screening taking place in ages 50–59 years, as primarily defined in the Finnish screening policy, have not been reported in detail from the randomised studies. In the Swedish trials, the screening effect was -16% (95% CI from -30% to 1%) among women started screening in age 50–59 and -33% (95% CI from -47% to -16%) among women started screening in age 60–69, respectively [21]. Unlike in the Finnish programme, those women who had started screening at age 50–59 were systematically screened also when they reached age 60 or more; therefore our current estimate (-8.6%) is not directly comparable. Among women aged 60–69 at diagnosis the effect from the current study was -16.6%, only about half of the impact reported from the above randomised studies. Concerning the latter comparison, it is likely that the deficit in the actual screening coverage in that age affects the Finnish rates.

A larger point estimate in effectiveness for screening women at age 50–69, in comparison with screening women only at age 50–59, is supported also by a recent report from the Copenhagen BC screening programme, analysis based on age at death (not age at screen as in our study) [3]. The average effect on the refined mortality in ages 50 to 79 at death was -25%; the corresponding average estimate at age 50 to 64 at death (where screening in age 50–59 should have primarily affected) as extracted from the report is -11% and at ages 65–79 at death (where screening in age 60–69 should have primarily affected) -30%. Our results on the refined mortality are in line with the findings from Copenhagen for the younger targeted age group, not with the findings for the older targeted age group.

An earlier study has attempted to estimate how big impact would have taken place in the 'routine' breast cancer deaths in Finland, if a biannual breast cancer screening programme was started in 1988 among women in ages 50 to 69 [22]. Screening coverage of 80% was assumed, as well as efficacy as reported from randomised trials. In ages 50 to 69 at death, the estimated average decrease in the population-based routine death rate from breast cancer was -8% during the first five years since the onset of the programme; and -14% and -19% during the next five-year periods. The estimate of the current study (-5.6%) was clearly smaller than the two latter estimates. It is likely that these findings of small impacts on the population-based rates in the current study can be explained largely by the rather narrow age group targeted historically in our country.

Impacts of breast cancer screening on incidence have not been investigated adequately for the service screening programmes. In randomised trials, controls usually became screened at the end of randomisation period (usually within 6–8 years since the onset) and therefore follow-up of incidence is affected by screening [1]. There is a unique report from a trial in Malmö, Sweden, where controls in ages 55–69 were not invited; suggesting about 10% excess in lifetime BC incidence attributable to screening [23]. There is another study, using a non-randomised design, suggesting much bigger excess incidence rates [24]. A long-term follow-up study of a recent routine screening programme and with correction for lead-time has suggested, however, that only rather small over-diagnosis of some 5% in relative terms might be expected [25]. A recent trend study from Finland, including a very long follow-up time since the last screen when screening was done in ages 50–59, reported no excess in the estimated cumulative incidence [26]. The current study suggests that if there is over-diagnosis in the Finnish BC screening programme, the relative estimate is small. Even a small excess risk in the breast cancer incidence may mean considerable numbers when contrasted with the numbers of deaths prevented; particularly when taking into account that the number of deaths prevented by screening in ages 50 to 59 only is rather small.

There was a consistent decrease in BC mortality since the mid-1990s in all the age groups studied. It was important to note also among women before age targeted for screening. This development may indicate an effect from improved treatments or from improved early diagnoses (stage migration) also outside organised screening. This trend was in a general agreement with the finding from US that developments in treatment and in early diagnosis in other fields of health care than in organised screening probably affect more in rather young age groups, say, 40 to 54, than in the older targeted ages of screening programmes [27]. This development could affect the numbers of deaths prevented and also the relative screening effect in the future.

One further limitation in estimating the tentative screening impact was that women in ages 40–49 (almost entirely unscreened), as well as women in ages 65–69 during the first few years of the programme, contributed to expectation without screening. We thought that inclusion of non-screened age groups was necessary to estimate screening effects (Table 3), because there were no unscreened regions in the country and otherwise we could not get an idea of developments during screening period – such as changes in background risk, changes in diagnostic activity or use of mammograms outside screening; or improvements in treatment – in absence of screening. For estimating the steady phase of the programme, when the prevalence screens take place mainly at age 50, we considered that the very low coverage of screening invitations below age 50 does not affect materially. Including experience in unscreened age groups during screening period seemed to alter the estimates meaningfully, compare the results obtained in the Tables 1 and 2 to those in the Table 3.

Irrespective of the decreasing trend since the mid-1990s, there was a rather large overall increase in the mortality rates in the long-term trend among women at age below 50 years. The increase in the background risk has been earlier reported to be among the highest in Finland [28] and it is the most likely explanation. Among young women the relative risk estimates may be imprecise, due to small numbers. One problem for the modelling was also that the baseline absolute mortality rate was much lower among the youngest age groups than among women in screening ages (Tables 1 and 2).

The incidence and mortality were modelled with the Poisson regression with numerical calendar time and polynomial functions of 5-year age group and synthetic cohort. When the orders of polynomials of age and cohort are included in the model, the changes in the incidence and mortality rates with age and cohort can be taken adequately into account. One must, however, remember carefully to check the sensibility and sensitivity of the model-based rates since this kind of modelling can lead to incredible predictions. We are looking forward to compare the current modelling results with other approaches for estimating incidence and mortality, especially using incidence-based mortality.

The effect of screening on incidence and mortality can be considered stable after 5 years since the beginning of the screening. Therefore the first five years with various effects on incidence and mortality were excluded from the basic descriptive analyses. One further problem when considering the national trends was that the Finnish programme was implemented gradually due to the randomised design during the implementation phase. Including or excluding that period did not materially affect the estimates.

In this study, information on screening was based mainly on the dynamic (open) information on the whole targeted population in ages 50–59. In older ages, 60–69 years, women were invited only partially or irregularly. Expected rates without screening were based on the patterns in time before the national screening programme, as well as on the experiences of non-screened age groups during screening era. As the whole population in the mainly targeted age was invited, there was no experience among non-screened in that age during the screening period. Using individual-level information on invited and screened during the current screening periods could still bring new information for evaluating screening effectiveness.

## Conclusion

In conclusion, the current study demonstrates that BC screening in Finland has been effective in reducing mortality from breast cancers. The impact is smaller than that observed in randomised trials. This may be explained at least partially by the rather narrow age group targeted; a great part of women in ages 60–69 that were included in the targeted populations in the randomised screening trials for breast cancer, were unscreened in our country. The results warrant considering whether screening should be targeted up to age 69. Considering rather small relative estimates, further research using individual-level information on the invited and screened can bring new information on the effectiveness of the screening programme.

## Competing interests

The author(s) declare that they have no competing interests.

## Authors' contributions

A.A. and S.H. have been responsible on performing the statistical analysis, T.S. has collected the screening information used in the current study, and T.H. has been involved in interpreting the results. All the authors have participated writing of this manuscript.

## Pre-publication history

The pre-publication history for this paper can be accessed here:



## References

[B1] International Agency for Research on Cancer (2002). IARC Handbooks of Cancer Prevention. Breast Cancer Screening.

[B2] Hakama M, Pukkala E, Heikkilä M, Kallio M (1997). Effectiveness of the public health policy for breast cancer screening in Finland: population based cohort study. Br Med J.

[B3] Olsen AH, Njor SH, Vejborg I, Schwartz W, Dalgaard P, Jensen MB, Tange UB, Blichert-Toft M, Rank F, Mouridsen H, Lynge E (2005). Breast cancer mortality in Copenhagen after introduction of mammography screening: cohort study. BMJ.

[B4] Gabe R, Duffy SW (2005). Evaluation of service screening mammography in practice: the impact on breast cancer mortality. Annals of Oncology.

[B5] The Swedish Organised Service Screening Evaluation Group (2006). Reduction in breast cancer mortality from organized service screening with mammography: 1. Further confirmation with extended data. Cancer Epidemiol Biomarkers Prev.

[B6] The Swedish Organised Service Screening Evaluation Group (2006). Reduction in breast cancer mortality from the organised service screening with mammography: 2. Validation with alternative methods. Cancer Epidemiol Biomarkers Prev.

[B7] Jonsson H, Nyström L, Törnberg S, Lenner P (2001). Service screening with mammography of women aged 50–69 years in Sweden: effect on mortality from breast cancer. J Med Screen.

[B8] Anttila A, Koskela J, Hakama M (2002). Programme sensitivity and effectiveness of mammography service screening in Helsinki, Finland. J Med Screen.

[B9] Parvinen I, Helenius H, Pylkkänen L, Anttila A, Immonen-Räihä P, Kauhava L, Räsänen O, Klemi P (2006). Service screening mammography reduces breast cancer mortality among elderly women in Turku. J Med Screening.

[B10] Törnberg S, Carstensen J, Hakulinen T, Lenner P, Hatscheck T, Lundgren B (1994). Evaluation of the effect on breast cancer mortality of population-based mammography screening programmes. J Med Screening.

[B11] Otto SJ, Fracheboud J, Looman CWN, Broeders MJM, Boer R, Hendriks JHCL, Verbeek ALM, de Koning HJ (2003). Initiation of population-based mammography screening in Dutch municipalities and effect of breast cancer mortality: a systematic review. Lancet.

[B12] Hakama M, Pukkala E, Söderman B, Day N (1999). Implementation of screening as a public health policy: issues in design and evaluation. J Med Screen.

[B13] Miller AB (2003). Is mammography screening for breast cancer really not justifiable?. Recent Results Cancer Res.

[B14] Olsen O, Gotzsche P (2001). Cochrane review on screening for breast cancer with mammography. Lancet.

[B15] Gotsche PC, Nielsen M (2007). Screening for breast cancer with mammography (review). Cochrane database of systematic reviews. The Cochrane Library.

[B16] Dean B, Pamilo M (1999). Screening Mammography in Finland – 1.5 Million Examinations with 97 percent Specificity. Acta Oncol.

[B17] oleman MP, Estève J, Damiecki P, Arslan A, Renard H (1993). Trends in cancer incidence and mortality. IARC Scientific Publications No 121, Lyon.

[B18] Sarkeala T, Anttila A, Forsman H, Luostarinen T, Saarenmaa I, Hakama M (2004). Process indicators from ten centres in the Finnish breast cancer screening programme from 1991 to 2000. Eur J Cancer.

[B19] Saarenmaa I, Salminen T, Varonen H, Fredriksson M, Sintonen H, Mäkelä M (2000). FinOHTAn raportti 16. Rintasyöpäseulonnan laajentamisen vaikutukset. Helsinki STAKES.

[B20] Immonen-Räihä P, Kauhava L, Parvinen I, Helenius H, Klemi P (2001). Customer fee and participation in breast cancer screening. Lancet.

[B21] Nyström L, Andersson I, Bjurstam N, Frisell J, Nordenskjold B, Rutqvist LE (2002). Long-term effects of mammography screening: Updated overview of the Swedish randomised trials. Lancer.

[B22] Hristova L, Hakama M (1997). Effect of screening for cancer in the Nordic countries on deaths, costs and quality of life up to the year 2017. Acta Oncologica.

[B23] Zackrisson S, Andersson I, Janzon L, Manjer J, Garne JP (2006). Rate of over-diagnosis of breast cancer 15 years after end of Malmo mammographic screening trial: follow-up study. Br Med J.

[B24] Jonsson H, Johansson R, Lenner P (2005). Increased incidence of invasive breast cancer after the introduction of service screening with mammography in Sweden. Int J Cancer.

[B25] Paci E, Warwick J, Falini P, Duffy SW (2004). Overdiagnosis in screening: is the increase in breast cancer incidence rates a cause for concern?. J Med Screen.

[B26] Seppänen J, Heinävaara S, Anttila A, Sarkeala T, Virkkunen H, Hakulinen T (2006). Effects of different phases of an invitational screening programme on breast cancer incidence. Int J Cancer.

[B27] Berry DA, Cronin KA, Plevritis SK, Fryback DG, Clarker L, Zelen M, Mandelblatt JS, Yakovlev AY, Habbema JDF, Feuer EJ, for the Cancer Intervention and Surveillance Modeling Netwrok (CISNET) Collaborators (2005). Effect of screening and adjuvant therapy on mortality from breast cancer. The New England J of Medicine.

[B28] Botha JL, Bray F, Sankila R, Parkin DM (2003). Breast cancer incidence and mortality trends in 16 European countries. Eur J Cancer.

